# Innovative Application of Fermented Red Bean Seeds in Constructing Foods with Increased Biological Activity

**DOI:** 10.3390/foods14010088

**Published:** 2025-01-01

**Authors:** Małgorzata Gumienna, Małgorzata Lasik-Kurdyś, Krystyna Szymandera-Buszka, Barbara Górna-Szweda, Dorota Walkowiak-Tomczak, Anna Jędrusek-Golińska

**Affiliations:** 1 Department of Food Technology of Plant Origin, Poznań University of Life Sciences, Wojska Polskiego 31, 60-624 Poznan, Poland; malgorzata.lasik@up.poznan.pl (M.L.-K.); barbara.gorna@up.poznan.pl (B.G.-S.); dorota.walkowiak@up.poznan.pl (D.W.-T.); 2 Department of Gastronomy Science and Functional Foods, Faculty of Food Science and Nutrition, Poznan University of Life Sciences, Wojska Polskiego 31, 61-624 Poznan, Poland; krystyna.szymandera_buszka@up.poznan.pl (K.S.-B.); anna.jedrusek-golinska@up.poznan.pl (A.J.-G.)

**Keywords:** aquafaba, fermented beans, bioactive compounds, sensory analysis, gastrointestinal tract, gut microbiota

## Abstract

Legumes are an interesting matrix for food production. The aim of this study was to develop functional plant-based snacks using fermented red bean (RBB) seeds enriched with the following additives: marjoram—RBM (2%); carrot—RBC (30%); and red beetroot—RBRB (15%). In the process of constructing the snacks, the focus was on the maximum use of the raw material, including aquafaba, to improve nutritional properties, sensory acceptability, and biological activity. The chemical composition, protein digestibility, antioxidant activity, and phenolic content were analyzed. In addition, the effect of the in vitro digestion process on biologically active compounds and their interactions with intestinal microflora was analyzed. Sensory analysis and consumer evaluation were performed. It was found that fermentation with lactic acid bacteria increased the content of total protein (by 2%), reducing the presence of substances (by 8%) and phenolic compounds (by 13%) in red bean seeds. Snacks with marjoram (RBM) showed the highest antioxidant activity (increase by 42%) and content of polyphenolic compounds (increase by 25%) compared to the basic variant (RBB). During digestion, the content of phenolic compounds and antioxidant activity reached the highest values in the last section of the digestive tract, i.e., in the large intestine, with RBM achieving the best results (5.61 mg GAE/g and 28.82 mg TE/g). The snack variants with red beetroot (RBRB) and marjoram (RBM) were rated the best by consumers. The results obtained confirm that the obtained snacks can be innovative products with health-promoting properties, and marjoram turned out to improve their properties, including antibacterial ones.

## 1. Introduction

Legumes are included in the Fabaceae family and belong to the group of coarse-seeded legumes. These are mainly annual plants grown for food. The most commonly consumed legumes include broad beans, chickpeas, beans, lentils, and soybeans [1,2,3].

Currently, in developed and developing countries, there is increasing interest in natural, unprocessed, and functional food. This has resulted in an increase in the consumption of legumes, which have a number of health benefits, as they are a very good source of protein and contain small amounts of fat. The presence of vitamins (mainly B vitamins), fiber, minerals (phosphorus, potassium), and biologically active ingredients such as polyphenols is also beneficial [3,4,5]. At the same time, they are low in calories and have a low glycemic index but do not cause a rapid increase in blood glucose, so they can be consumed by people with diabetes.

Thanks to the content of compounds with antioxidant activity, legume seeds, including red beans, have a confirmed positive effect in the prevention of the so-called civilization diseases, which include, inter alia, diet-related diseases (overweight, obesity), cardiovascular diseases, and cancer [4].

The main chemical component of legumes is carbohydrates. Their percentage share is about 50%. The saccharide that makes up the majority is starch. They also contain small amounts of dietary fiber, which is important due to its health-promoting properties [1,2,4].

Legume seeds are a rich source of protein—their content can range from 20 to 44%. Lipids in legumes are found in insignificant amounts—these values range from 0.5 to 2%. A few oily species, such as soybeans and lupins, are characterized by a high lipid content. The predominant fatty acids are linoleic and oleic [1].

In addition to beneficial nutrients, legumes contain so-called antinutrients, such as trypsin and chymotrypsin inhibitors, which can adversely affect consumers, especially from the perspective of increasing the consumption of protein products [5]. Therefore, it is advisable to subject legume seeds to technological processes, including fermentation, to reduce the content of oligosaccharides [6] and increase the content of functional compounds, such as vitamins (especially B2 and B12), antioxidants, and other food ingredients [6,7].

The use of legumes to design a variety of food products with nutritional properties and beneficial effects on health, such as pasta, bread, soups, or snacks [8], is currently the center of interest, especially for the young generation (vegans, vegetarians, and flexitarians) looking for substitutes for animal protein. In vegan products dominated by other additives, such as cold cuts, bean lard, plant-based frankfurters, etc., where there is no noticeable so-called “bean” aftertaste, such a product is acceptable to young consumers [8,9]. A good example of the use of legumes in baking is research conducted on the replacement of soy flour with fermented and non-fermented broad bean flour in the production of gluten-free bread. These studies showed that the fermentation of flour caused a significant increase in the content of essential amino acids and the nutritional value of the obtained bread. At the same time, no changes in the sensory properties of the obtained bread were found in relation to the bread obtained from unfermented flour and soy flour [10]. An additional aspect relating to the favorability of the increasing impact of proteins obtained from legumes in the market of animal protein substitutes is the possibility of using by-products occurring during production, e.g., okara (soy pomace), which is formed after the production of tofu from soybeans or aquafaba after soaking in liquid during the production of hummus [9,11,12,13]. Aquafaba is a by-product of soaking or cooking legumes used to make vegan mayonnaise [12,13]. The upcycling food market is expected to grow by 5% by 2029 and food waste is expected to reduce by 50% by 2030 [9,13].

However, the use of legumes, mainly broad beans or beans, for the production of food based on them is associated with tastes and smells that are not always acceptable to consumers. The sensory properties of the products need to be refined to meet the expectations of increasingly demanding consumers [10,14].

Therefore, the aim of this study was to develop a waste-free production process for wafer-type vegetable snacks from fermented red bean seeds with improved sensory acceptability and nutritional properties. For this purpose, in addition to fermented whole red bean seeds, aquafaba was used in the production of wafers, and marjoram and vegetables (carrots, red beets) were added. This not only enriched the products with bioactive ingredients but also affected their taste and aroma.

## 2. Materials and Methods

### 2.1. Raw Material

Beans (*Phaseolus vulgaris* L., cultivar Red Kidney) were obtained from Polish crops and purchased from the Seed Headquarters in Nochowo, Poland. Dried leaves of marjoram (*Majorana Majorana* L.) and vegetables—the common carrot (*Daucus carota* L., cultivar Karotina) and red beetroot (*Beta vulgaris* L., cultivar red ball)—were purchased from a local market (Poznań, Poland).

### 2.2. Production of Plant-Based Snacks

At the initial stage of the process, the seeds were soaked and boiled in the same water for about 30 min. Then, the seeds were cooled and, together with the liquid residue (aquafaba), ground until a structure with a particle size of 0.5 to 0.8 mm was formed. Water was added to the mass prepared to obtain the final moisture content of about 50%. The mass prepared this way was subjected to the lactic fermentation process with the probiotic bacteria *Lactobacillus plantarum* (LP commercial strain purchased: www.serowar.pl). The mass was fermented at 37 °C for 20 h based on the modified methodology by Gumienna et al. [15]. After the fermentation process, the semi-finished product was mixed with the additives inulin (3%), salt (0.5%), marjoram (2%), grated cooked (30 min) red beetroot (15%), and grated raw carrot (30%). Then, circular wafers with a diameter of 10 ± 1 cm and a weight of 70 ± 5 g were formed and freeze-dried (type Alpha 2–4 LD plus, Christ, Osterode/Harz, Germany) under a pressure of 0.12 mbar for 48 h, with an initial freezing process at 80 °C for 24 h. Finally, the following 4 variants of products were prepared:

- Snacks such as red bean wafers with inulin 3%, salt 0.5%—basic variant (RBB);
- Snacks such as red bean wafers—basic variant + marjoram (2%) (RBM);
- Snacks such as red bean wafers—basic variant + carrot (30%) (RBC);
- Snacks such as red bean wafers—basic variant + red beetroot (15%) (RBRB).

### 2.3. Chemical Composition

The raw materials and the plant-based snacks obtained were analyzed in regards to their dry matter content [16], total protein content [17], reducing sugars with DNS (3,5-dinitrosalicylic acid), according to the Miller method [18], and soluble protein content by the Lowry method [19]. Protein digestibility was determined using the in vitro pepsin-pancreatin method [20].

### 2.4. In Vitro Digestion Process

The process of digestion of the obtained plant-based snacks was carried out in an in vitro model of the gastrointestinal tract simulating the stages of the “stomach” with the “duodenum”, “small intestine”, and “large intestine”. The digestion was conducted in a glass bioreactor equipped with 4 inlets, allowing the introduction of the pH electrode, programming of the active acidity, dosage of biochemical agents, and appropriate media, as well as the collection of analytical samples. Samples for the in vitro digestion process were prepared by taking 23 g of products and dissolving them in demineralized water to a volume of 230 mL. The parameters of the digestion process were selected based on our previous investigations [21,22]. The entire process was carried out by the methodology described by Kowalczewski et al. [23]. During the process, the total polyphenol content, antioxidation potential, reducing sugars, soluble protein, and β-glucuronidase and β-glucosidase activity were determined.

### 2.5. Preparation of Samples from Red Bean for the Analysis of Biologically Active Compounds

The sample before and after fermentation was ground in a laboratory mill (Witko, Łód’z, Poland). A total of 0.5 g of the homogeneous sample was weighed into a centrifuge tube containing 10 mL of a 70:30 acetone/water extraction mixture. It was shaken for 60 min on a rocker shaker and centrifuged at 4125× *g* for 7 min. The supernatant fluid (extract) was decanted and used to perform the following analyses. The procedure for conducting the extraction process was performed based on the methodology described in the Szymandera-Buszka et al. [14].

Preparation of the samples for analysis after the in vitro digestion process included the following: The extracts were prepared in a 10 mL tube to which 3 mL of digested content and 7 mL of acetone (≥99%) were added to obtain a 70% concentration. The tube was shaken for 60 min on a rocker shaker and centrifuged at 1700× *g* for 15 min. The supernatant (300 μL) was used for the total polyphenols and antioxidative activity analyses.

#### 2.5.1. Total Polyphenols Content

The total polyphenol content was measured using the modified Folin–Ciocalteu method [24], and its values were estimated from a standard curve of gallic acid. The result was expressed as mg gallic acid equivalent (GAE g^−1^ dry matter) (Sigma-Aldrich, Munich, Germany) and, in the case of the digestion process, in mg per g of digested products [21]. All results were corrected for the presence of phenols in the pancreatin/bile salts mixture.

#### 2.5.2. Antioxidant Activity

The total antioxidant capacity (TEAC) was determined against the ABTS reagent (2,20-azinobis-(3-ethylbenzothiazoline-6-sulphonic acid)) (Sigma-Aldrich, Munich, Germany) according to the method described by Re et al. [25] under the methodology provided by Szymandera-Buszka et al. [14]. The results of the TEAC assay were expressed as the capability of antioxidants to scavenge ABTS radicals relative to that of Trolox (a water-soluble vitamin E analog) and was reported as mg TE·g^−1^ dry matter (Sigma-Aldrich, Munich, Germany) and, in the case of the digestion process, it was reported as mg TE/g of the digested products [21].

### 2.6. β-Glucuronidase and β-Glucosidase Activity

The determination was carried out based on a modification of Djouzi and Andrieux [26] and Kapnoor and Mulimani [27]. The sample was prepared by taking 200 μL of content from the bioreactor, to which 1500 μL of phosphate buffer (pH 7.0) and NaCl (0.1 M) were each added. The samples were shaken for 1 h, then centrifuged for 15 min, 3000 rpm, obtaining a supernatant. A total of 200 μL of p-nitrophenyl-β-D-glucuronide (for β-glucuronidase (EC 3.2.1.31) (Sigma-Aldrich, Munich, Germany) or p-nitrophenyl-β-D-glucopyranoside (for β-glucosidase (EC 3.2.1.21) (Sigma-Aldrich, Munich, Germany) was added to 200 μL of the supernatant, followed by incubation for 2.5 h at 40 °C. The reaction was stopped by the addition of 2000 μL of sodium carbonate. The absorbance was measured at 420 nm. Enzymatic activity was expressed as μM of the product (p-nitrophenol) formed in 1 min per gram of digested content (U per g digested content).

### 2.7. The Intestinal Microflora

To control the influence of the conditions prevailing in the gastrointestinal tract on the growth of microorganisms, control inoculations were made after 2 h from the moment (pH 7.4, small intestine) of introducing microorganisms into the environment and at the moment of the termination of the digestion process (after 21 h). The intestinal microflora isolated from the faecalis of a mature person was introduced into the experimental model. The determined groups of the microorganisms included *Enterobacteriaceae* (MacConkey selective medium—Sigma Aldrich, Saint Louis, MO, USA), *Lactobacillus* (MRS agar medium—Sigma Aldrich, Saint Louis, MO, USA), *Enterococcus* (substrate—agar with kanamycin, esculin, and sodium azide), and *Bifidobacterium* (Garche medium—Sigma Aldrich, Saint Louis, MO, USA). Inoculated media were incubated in anaerobic conditions depending on the determined group of microorganisms for 48 to 72 h at 37 °C [22]. The Koch’s plate method determined the number of viable bacterial cells [23].

### 2.8. Sensory Analysis

Sensory analysis studies were conducted in the sensory analysis laboratory [28] at the Department of Gastronomy Science and Functional Foods, Poznan University of Life Sciences, Poland.

This study was conducted in accordance with the Declaration of Helsinki, and the protocol was approved by the Ethics Committee of Poznan University of Medical Sciences (Project identification code no. 757/13). This research was conducted with the participation of consumers who declared their voluntary, written consent to participate in the study. Participants were informed about the study’s aim and that their participation was entirely voluntary; therefore, they could stop the analysis at any point, and their responses would be anonymous. Additionally, these data were pseudonymized. A code was assigned to each person.

The 10 g (one wafer) samples were served in plastic containers (200 mL) with lids.

The samples were coded with three-digit numbers, and the serving order was random (the program ANALSENS—v.5.0; Sopot; Poland). Unsweetened black tea (≈45 °C) was served to neutralize the taste of the samples. Sensory analysis among consumers was conducted on 240 people aged 20–42 (who consume crisp bread at least twice a month). Women constituted 58% of the population analyzed. Consumers tested the desirability of the color, taste, aroma, texture, and overall desirability. A 10 cm hedonic graphic scale was used, with the following margin denotations: undesirable to highly desirable. All consumers rated all samples in one session.

The sensory profiling of taste was conducted by an 8-member tested panel. A total of 9 descriptors were adapted for aroma (essential oil, herbal, starch, lemon, bitter, strange, sour, fermentation, and broth) and 9 descriptors for taste (essential oil, herbal, sour, salty, sweet, starch, broth, bitter, and strange). The intensity of each descriptor was determined using a 10 cm linear scale with appropriate margin descriptions. Uniform margin denotations were applied for the attributes: “undetectable to very intensive”. All samples were assessed in two independent replications.

### 2.9. Statistical Analysis

The results were analyzed statistically with the STATISTICATM PL 13.3 software (StatSoft, Tulusa, OK, USA). All measurements were studied using a one-way analysis of variance independently for each dependent variable. Post hoc Tukey honest significant difference (HSD) multiple comparison tests were used to identify statistically homogeneous subsets at α = 0.05.

For the overall evaluation of the differences and similarities in the sensory profiles, consumer analysis, and content of active compounds of the tested samples, analysis of the main components (PCA—principal component analysis) was used. Hypotheses were tested at α = 0.01.

## 3. Results

### 3.1. Chemical Content and Biologically Active Compounds of Plant-Based Snacks During or After Biotechnological Treatment

**Figure 1:**
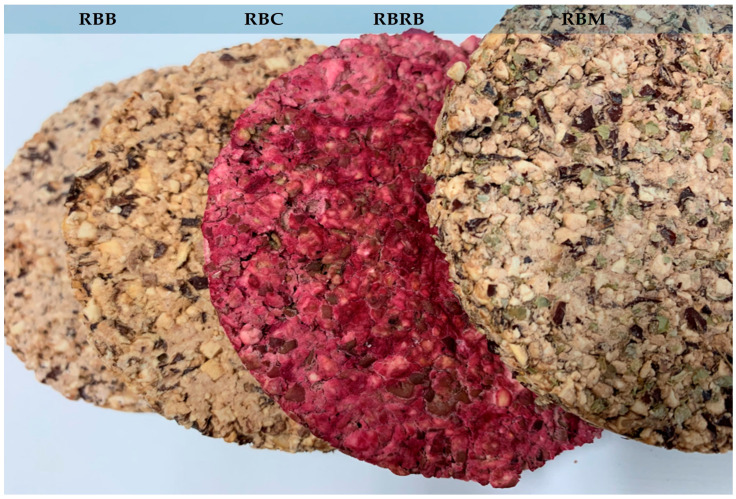
RBB—snacks such as red bean wafers with inulin 3%, salt 0.5% basic variant; RBM—snacks such as red bean wafers basic variant + marjoram (2%); RBC—snacks such as red bean wafers basic variant + carrot (30%); RBRB—snacks such as red bean wafers basic variant + red beetroot (15%).

To construct plant-based snacks in the form of wafers with functional properties, red bean seeds were subjected to biotechnological processing to the four product variants obtained (Table 1, Figure 1), where, on the one hand, a by-product formed during hydrothermal processing of bean seeds (aquafaba) was used, and on the other hand, additives were used to enrich the taste and affect the content of biologically active compounds in the products obtained (Table 2). During the research, red bean seeds and snacks made from them were characterized, considering the process of lactic acid fermentation. Samples were analyzed for their content of dry matter, reducing substances, protein (soluble and total), protein digestibility, the total content of polyphenolic compounds, and their antioxidant activity (Table 1). The effect of lactic acid fermentation on the nutritional components of red bean seeds was confirmed. Protein digestibility increased significantly from 29% to about 79%, the total protein content increased after fermentation (AFRB) by 2%, reducing substances by 8%, and the soluble protein content was decreased by about 53% compared to the sample before the process (BFRB). Moreover, after fermentation, a significant increase was noted in both the total content of phenolic compounds (13%) and their antioxidant activity (72%).

The highest increase in the phenolic compound total content and activity was noted in the case of plant-based snacks with the addition of marjoram (RBM) by 25% and 42%, respectively (Table 1). The obtained RBM snacks, in relation to pure snacks without any additive (RBB), were characterized by a high content of soluble protein—an increase of 22%, total protein—an increase of 6%, and reducing substances—an increase of 84%. No effect on the total content of polyphenols was found in the snacks obtained with the remaining additives of carrot (RBC) and red beetroot (RBRB). At the same time, in the case of antioxidant activity, an increase was noted at the levels of 14 and 18%, respectively. For the remaining nutrients, an increase in soluble protein was found as follows: by 5% for RBC, 6% for RBRB, 8% for total protein for RBC, 5% for RBRB, and reducing substances by 22%. In the case of RBC snacks, the highest content of reducing substances was found among all the products obtained, four times higher than the initial content in RBB snacks. The freeze-drying process allowed for obtaining products with a very high dry matter content of 90–91% and high protein digestibility from 80 to 83%, the highest for RBM and RBRB products (Table 1).

Table 2 presents the values of dry matter content, reducing substances, soluble proteins, total proteins, polyphenols, and antioxidant activity of the additives used for plant-based snacks. Dried marjoram is characterized by the best parameters in terms of protein content (116.09 mg·g^−1^ dm), polyphenols (29.20 mg·g^−1^ dm), and antioxidant activity (87.73 mg·g^−1^ dm), which is why it became an excellent additive with the participation of which the obtained RBM products were characterized by an increased nutritional value (Table 1). On the other hand, the highest content of polyphenols (80.48 mg·g^−1^ dm) was noted in cooked red beetroot, and antioxidant activity was lower than in the case of marjoram (55.35 mg·g^−1^ dm). However, these values did not translate into their level in the final product (Table 1). Raw carrots have the highest content of reducing substances—170.22 mg g^−1^ dm among all used additives. On the other hand, the by-product aquafaba (after hydrothermal treatment of red bean seeds) is an additional source of protein (12.60 mg g^−1^) and reducing substances (5.02 mg g^−1^), enriching the final product.

### 3.2. Sensory Quality of the Obtained Plant-Based Snacks

**Figure 2:**
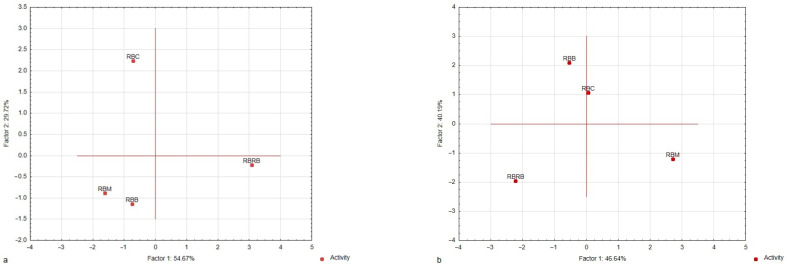
Map of the variants of plant-based snacks (red bean wafers) with marjoram, carrot, or red beetroot and the control sample (without marjoram, carrot, or red beetroot) into factors (F1 × F2). Case–factor coordinate plots based on the attributes of (**a**) aroma profiles and (**b**) taste profiles (PCA analysis).

The sensory profiling developed snacks and determined the perception of their aroma and taste descriptors intensity (Figure 2a,b, Table S1). The sensory profiling confirmed nine descriptors for aroma (essential oil, herbal, starch, lemon, bitter, strange, sour, fermentation, and broth) and nine for taste (essential oil, herbal, sour, salty, sweet, starch, broth, bitter, and strange). The sensory profiling did not confirm the perceptible bean’s aroma and taste. Principal component analysis (PCA) was used to study the relationship between the aroma and taste attributes of the characteristic sensory profiles of analyzed plant-based snacks (variables) and to derive factors according to which these variables can be classified (Figure 2a,b). The PCA showed that the first two factors (F1 and F2) were the most important elements, explaining variations in the data. They explained approximately 84% of the variance for aroma and 87% for taste, so they were selected for data interpretation. The absolute values of the factor coordinates of variables showed a relationship between the factors and the sensory attributes of the analyzed samples (Table 3). For the aroma attributes of both products, the first factor (F1) was most strongly related to essential oil, herbal, and starch aromas, and the second factor (F2) was to bitter and strange aromas. For the taste attributes of the samples, F1 was the most strongly related to sweet, broth, and bitter tastes, and F2 to herbal, sour, and salty tastes. For the aroma descriptors, a projection of the variants of samples on the factor-plane F1 × F2 (Figure 2) confirms that the RBC, RBM, and RBB samples were plotted to the left side of the F1 axis (i.e., they have negative coordinate values for F1).

These samples were characterized by the highest fermentation and essential oil aroma intensity. It was found that the RBRB samples were plotted to the right side (positive coordinate values for F1). The highest starch aroma intensity and lowest fermentation and essential oil aroma intensity characterized the aroma profiles of these samples (Table S1). For the taste descriptors, the projection of the snack variants on the factor-plane F1 × F2 confirms that the RBRB samples were plotted to the leftmost side of the F1 axis (i.e., they have negative coordinate values for F1). The RBM samples were plotted to the right side of the F1 axis (i.e., they have positive coordinate values for F1). These samples were characterized by the highest intensity of essential oil, herbal, and bitter tastes. The RBM and RBRB samples were plotted to the left side of the F2 axis (i.e., they have negative coordinate values for F2).

**Figure 3:**
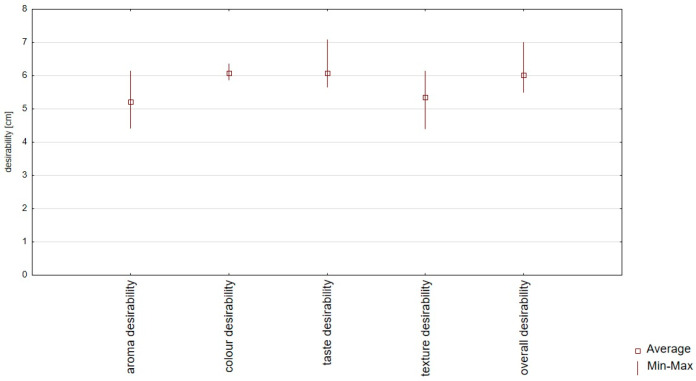
Box plot diagram of sensory consumer analysis (color, taste, aroma, texture, and overall desirability) of plant-based snacks (red bean wafers) with marjoram, carrot, or red beetroot and the control sample (without marjoram, carrot, or red beetroot).

**Figure 4:**
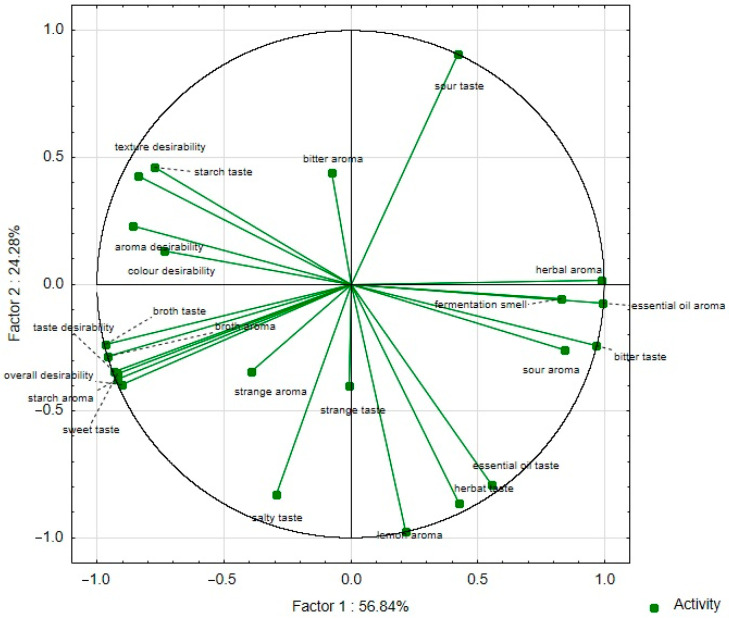
Principal component analysis (PCA) of the loadings plot of the sensory descriptors and consumer analysis parameters of plant-based snacks (red bean wafers) with marjoram, carrot, or red beetroot and the control sample (without marjoram, carrot, or red beetroot) into factors (F1 × F2).

Consumer evaluation analysis proved high color, taste, smell, texture, and overall desirability values, ranging from 4.42 to 7.07 points (Table S2 and Figure 3). The results of the sensory desirability analysis confirmed that there were no significant differences between all samples (Table S2). The highest positive correlation between the overall desirability and taste desirability changes (r = 0.99) was confirmed, and the lowest positive correlation between the overall desirability and texture desirability changes (r = 0.53) was confirmed (Table 4, Figure 4). The statistical analysis (Table S2) of aroma desirability confirmed the negative correlation between the intensity of essential oil (r = 0.916) and fermentation (r = 0.814) and the positive between starch aroma (r = 0.978). The statistical analysis of taste desirability confirmed the negative correlation between the intensity of sour (r = 0.704) and bitter (r = 0.812) taste and the positive between sweet (r = 0.981) taste.

The consumer analysis did not confirm significant differences in sensory desirability to the attributes of all samples (Table S1). However, the sensory profile analysis showed significant differences. Therefore, the sensory profile analysis results were used to analyze the correlation between the chemical indicators of the designed snacks and the intensity of sensory descriptors. The statistical analysis (Table S3) of the correlation coefficients between the intensity of the sensory descriptors and chemical parameters of the analyzed snacks confirmed the positive relationship between the protein content and salty taste (r = 0.98) and the negative relationship between sour taste (r = 0.97). The statistical analysis confirmed the positive relationship between the total polyphenols content and salty taste (r = 0.97) but did not confirm the relationship between the total polyphenols content and bitter taste (r = −0.15).

### 3.3. Effect of In Vitro Digestion on Total Polyphenols and Antioxidant Activity

Table 5 presents changes in the total content of the phenolic compounds and their activity during the digestion process in the in vitro digestive tract model of the plant-based snacks obtained from fermented red bean seeds (RBB, RBM, RBC, and RBRB). During the digestion process of the products obtained from legume seeds, changes occurring at individual stages of the process in the content of phenolic compounds and their antioxidant activity are crucial in considering their nutritional value or bioavailability. The digested RBB, RBM, RBC, and RBRB snacks showed significant differences in the initial content of phenolic compounds and antioxidant activity. The highest parameters were characteristic of the RBM snack, 2.85 mg GAE·g^−1^ and 5.92 mg TE·g^−1^ of digested content, and the lowest RBRB content, 1.29 mg GAE·g^−1^ and 1.99 mg TE·g^−1^. At the “stomach” stage, where the acidic environment dominates (pH 2.0), significant increases in the contents of polyphenols in RBB by 11%, RBM by 15%, and RBRB by 80% were noted in the analyzed samples; only in the RBC sample was there no significant change noted. However, after “duodenum” (pH 7.4), the analyzed content of the phenolic compounds in all samples decreased from 9% (RBRB) to 54% (RBM). In the case of the antioxidant activity determined at the “stomach” stage, a significant decrease was noted in the samples: RBB (70%), RBM (3%), and RBRB (22%), while in the RMC sample, an increase (52%) was noted. At the “duodenum” stage, an increase in the activity analyzed was noted, however, these differences were not significant to the samples before digestion. The smallest changes in antioxidant activity fluctuations at these stages of the digestion process were noted in the RBM samples, from 3% to 13%, which suggests that phenolic compounds released as a result of the digestion process, despite their 54% reduction, show high antioxidant activity.

At the stage of the process where the digested content is exposed for 2 h to intestinal microflora (“small intestine”), the content of phenolic compounds increased in RBB (34%) and RBM (13%); only during the digestion of RBC and RBRB snacks, the changes do not have a significant effect on the content of the analyzed compounds. At this stage of the process, an upward trend was noted for antioxidant activity in all analyzed samples. The highest activity was again characterized by the RBM sample (15.84 mg TE g^−1^).

In the final section of the digestive tract (“large intestine”), after 18 h of digestion, the content of phenolic compounds and antioxidant activity reached the highest values, especially for the RBB snacks: 3.92 mg GAE·g^−1^ and 24.10 mg TE·g^−1^, and RBM: 5.61 mg GAE·g^−1^ and 28.82 mg TE g^−1^ (Table 5).

The results obtained indicate that the digestion process affects the content of phenolic compounds and antioxidant activity. The recorded increase in these parameters in the final section of the digestive tract indicates the key role of intestinal microflora in their biotransformation during the process. Plant-based snacks with added marjoram (RBM) are distinguished by the highest antioxidant activity and content of phenolic compounds at all stages of the digestion process, which may indicate their high health-promoting potential.

### 3.4. Changes in Reduced Substance and Soluble Protein During Digestion

During the digesting process of plant-based snacks in the digestive tract model, changes in the content of reducing substances and soluble protein were also analyzed (Table 6).

During the process, it was found that in the initial stages of the process (“stomach’, “duodenum”), the content of reducing substances and soluble protein significantly increases. In the case of the base snack without additives (RBB), a 9-fold increase in the content of reducing substances was noted in the initial sample; in RBM, a 2-fold increase; and in the snack with the addition of red beetroot (RBRB), as much as 12-fold. Only in the case of the snack with the addition of carrot (RBC) was an increase of about 1.2-fold noted. On the other hand, the values of soluble protein increased 5-fold for the RBB, RBC, and RBRB samples, and in the RBM snack, a 6-fold increase was noted. Such an intensive increase in the content of reducing substances and soluble protein is related to the enzymatic activity of the enzymes introduced into the digestion process, which reflects the conditions prevailing in this section of the human digestive tract.

In the key stages of the digestion process involving intestinal microflora (“small intestine” and the beginning of the “large intestine”), the highest values of reducing substances were recorded for the RBB (97.16 mg·g^−1^) and RBM (90.62 mg·g^−1^) snacks. In the case of the RBC, the content of the analyzed compounds after 2 h of digestion in the “small intestine” was significantly lower than at the “duodenum” stage by about 50%. A similar trend was observed in the case of the RBRB snack (85%). However, a different digestion mechanism was found for soluble protein. After the “stomach” stage and the intensive protein decomposition process (increase in soluble protein value), regardless of the digested snack variant, digestion with the participation of intestinal microbiota (beginning of the “large intestine”) resulted in the achievement of the highest soluble protein values in the process (RBB: 330, RBM: 91, RBC: 124, and RBRB: 111 mg·g^−1^). After 18 h of digestion in the “large intestine”, a further increase in soluble protein was noted for the analyzed samples, except for the basic sample (RBB), where the content decreased from 330 mg·g^−1^ to 204 mg·g^−1^. The results obtained reflect the intensity of the digestion process and indicate the important role of intestinal microflora in the metabolism of these compounds. This thesis is confirmed by the results obtained after the digestion process for the determined values of reducing substances, where, as a result of the action of intestinal microflora, these values decreased, respectively, RBB: 5-fold, RBM: 2-fold, and RBRB: 6-fold, and only in the case of the RBC sample did they increase 3-fold (Table 6).

The increase in the values of reducing substances and soluble proteins at individual stages of the digestion process indicates a close interdependence of the intestinal microbiome, its activity, the plant base, and additives, with the participation of which the snack was composed. Various additives used in the technological process of making snacks (RBB, RBM, RBC, and RBRB) showed different susceptibility to the conditions prevailing in the digestive tract model during the conducted process, indicating differences in their functional properties.

### 3.5. Effects of β-Glucuronidase and β-Glucosidase Activity

Table 7 presents the results of the analyses of β-glucuronidase (EC 3.2.1.31) and β-glucosidase (EC 3.2.1.21) activity during the digestion process of the plant-based snacks RBB, RBM, RBC, and RBRB, taking into account those stages in which intestinal microflora was added. It was found that in both analyzed cases, regardless of the type of digested snack, both β-glucuronidase and β-glucosidase activity showed a decreasing trend.

The most significant changes during the digestion process occurred during the digestion of snacks with the participation of marjoram (RBM). In the case of β-glucuronidase, the activity of this enzyme after 18 h of digestion in the large intestine showed the lowest activity. The digested content’s initial value of 0.188 U·g^−1^ finally reached 0.042 U·g^−1^. Similar observations were made regarding the activity of β-glucosidase, which was determined during the digestion process of the same snacks (RBM). The initial activity was at 0.252 U·g^−1^ of the digested content, while after 18 h of digestion, it was 0.112 U·g^−1^ (Table 7). Thus, the snack with the addition of 2% marjoram is characterized by the most dynamic changes among the analyzed variants. However, it should be emphasized that the determined enzymatic activity in the case of each variant showed a significant decrease in activity. This suggests that the digested content included compounds—inhibitors of enzymatic activity that affected not only the enzymatic activity but also intestinal microflora and their metabolism.

### 3.6. Effect of Gut Microbiota

During the in vitro digestion process, the numbers of individual strains of microorganisms were determined at the stages from the introduction of microorganisms (“small intestine” after 2 h at pH 7.4) to the final stage (“large intestine” after 18 h at pH 8.0). The results are presented in Table 8.

It was observed that the base snacks (RBB) at the last stage of the in vitro digestion process significantly affected the growth of bacteria of the genera *Lactobacillus, Bifidobacterium,* and *Enterococcus*. The numbers of bacteria determined at the last stage of the process were 11.70 log 10 cfu·mL^−1^, 11.67 log 10 cfu·mL^−1^, and 11.64 log10 cfu·mL^−1^, respectively, which indicates a positive effect of the snack composition on the intestinal microbiota. However, a different trend was noted for bacteria of the genus *Escherichia coli*. During the process, no significant changes were noted in the number of determined bacteria.

In the case of the RBM snack (marjoram added), a significant increase was noted for bacteria of the following genera: *Lactobacillus, Bifidobacterium*, and *Enterococcus*; the final number of bacteria was determined at levels of 10.2 log 10 cfu·mL^−1^ and 7.55 log 10 cfu·mL^−1^, respectively, and was significantly lower to the base sample. On the other hand, for *E. coli* bacteria, inhibition of the growth of this microorganism was noted. From the initial level of 6.68 log 10 cfu·mL^−1^, at the last stage of the process, the number of bacteria was determined at the level of 3.95 log 10 cfu·mL^−1^.

The digestion process of the RBC (with carrot) and RBRB (with beetroot) snacks in the case of determining the number of bacteria of the genera *Lactobacillus, Bifidobacterium,* and *Enterococcus* did not show an effect limiting the growth of these microorganisms. After 18 h of digestion, the number of bacteria determined ranged from 9.76 to 10.50 log 10 cfu·mL^−1^. The effect of inhibiting bacterial growth, similarly to the RBM snack, was noted in the case of *E. coli*, and so for the RBC snack, the number of bacteria after the digestion process was at the level of 5.94 log 10 cfu·mL^−1^, and for RBRB, it was 7.28 log 10 cfu·mL^−1^ (Table 8).

Based on the results obtained, it can be concluded that the obtained RBB, RBM, RBC, and RBRB snacks, on the one hand, have a positive effect on the growth of lactic acid bacteria, with the best base snack (RBB), and on the other hand, the best potential antibacterial properties against pathogens such as *E. coli* are demonstrated by the RBM snack.

## 4. Discussion

The results of this work confirmed that red bean seeds can be a valuable raw material for constructing plant snacks. As a result of the applied biotechnological process, snacks were made based on fermented legume seeds with additives affecting the composition, taste, and aroma (marjoram, carrot, and red beetroot).

Previous studies [4,5,7,8] also showed the possibility of using red bean seeds to construct functional food, such as food with an increased content of biologically active compounds, which provides the possibility of obtaining products ready for direct consumption that are intended for people looking for natural substitutes for animal protein [29].

The use of the fermentation process with the participation of lactic acid bacteria affects the chemical composition and nutritional value of the raw materials used [30]. In this study, it was shown that fermentation significantly affected the digestibility of proteins (from 29 to 80%) of red bean seeds. In the studies, before the fermentation process, heat treatment of bean seeds was used for 30 min. Heat treatment, such as cooking or extrusion, increases the digestibility of protein in vitro, and the intensity of this digestibility is influenced by the duration of the heat treatment (cooking) [8,31].

The fermentation process used in this study affected the total protein content in red bean seeds, which increased by 2% compared to the seeds before fermentation (143.40 mg g^−1^). As reported by Kimothi and Dhaliwal [5], this is influenced by the enzymes produced by lactic acid bacteria, responsible for the breakdown of protein into more easily digestible peptides [5]. The fermentation process also affects the content of phenolic compounds and their activity [32,33], which may lead to better health-promoting properties and sensory values of food products. The studies conducted in this study indicate that fermentation caused a significant increase in both the total content of phenolic compounds (13%) and their antioxidant activity (72%). Li et al. [33], in their studies, also showed that fermentation increases the content of phenolic compounds in powders obtained from beans. In addition, microbial α-amylase and protease produced during fermentation can contribute to the release or synthesis of new phenolic compounds [32], improving the nutritional value [29] as well as the sensory properties of the final products to acceptable standards for consumers, like taste, aroma, and texture [14].

To improve the structure and reduce the addition of water for the fermentation process, aquafaba obtained after hydrothermal treatment (dipping and cooking) was used. Aquafaba’s addition provides the possibility of making a food product using a by-product, following the technology of waste-free food production. Moreover, the choice of the method of preservation of plant-based snacks (e.g., freeze-drying process) is not without significance when maintaining the activity of bioactive compounds and preserving the nutritional value of legumes [34,35,36,37], which this study confirmed.

The results obtained at this stage confirmed that the additives used, such as marjoram, carrot, and red beetroot, significantly affect the content of nutrients in the obtained plant snacks. The best results were obtained in the case of snacks with the addition of 2% marjoram (RBM), where the highest increase in the total content of phenolic compounds and their activity was noted by 25% and 42%, respectively. Moreover, marjoram, as shown in this study, was characterized by a high content of protein (116.09 mg·g^−1^ dm), polyphenols (29.20 mg·g^−1^ dm), and antioxidant activity (87.73 mg·g^−1^ dm). Therefore, it became an excellent additive, with the participation of the obtained RBM products characterized by an increased nutritional value, and can be accepted in the consumer assessment [38]. Our study confirmed that the samples with marjoram (RBM) had the highest intensity of sweet, broth, and bitter tastes. It was also confirmed that in the case of the RBRB samples, the dominant flavor noted was the intense herbal, bitter, and essential oil tastes. The bitterness sensation, as suggested by Drewnowski and Gomez-Cameros [39] and Soares et al. [40], may be related to the high content of phenolic compounds. However, the statistical analyses of our data did not confirm the relationship between the total content of polyphenols and the taste of bitterness and essential oils, especially for snacks with the addition of marjoram (RBM). The designed snacks had a bitter taste at a low intensity. Previous studies also confirm the lower volatility of aromatic compounds in extruded products [41]. Other studies also confirm the low intensity of bitter tastes in meat products by adding marjoram extract (lower than thyme) [14].

The high sensory appeal of meat products with marjoram extract, despite the high intensity of spicy and essential oil tastes in their sensory profile, may explain the popularity of this spice. Consumers who often use marjoram to season dishes may grow used to its characteristic aroma and taste [42,43]. In the evaluation of the aroma profile of plant-based snacks, it was found that the intensity of fermentation aroma and essential oil dominated the RBC, RBM, and RBB snacks. In contrast, the RBRB snacks were characterized by a higher intensity of starch aroma and a low intensity of fermentation and essential oil.

Other studies confirmed that oily and spicy tastes and aromas usually do not originate from a single compound but result from the concentration of various compounds in plant products and foods, including terpenes and aldehydes. Many of these substances are volatile [44]. Future studies suggest examining this tendency by other experiments, such as using instrumental methods. Moreover, our study confirmed the highest positive correlation between overall desirability and changes in taste desirability, confirmed by previous studies [14,38]. The developed recipe for plant-based snacks, such as waffles made from fermented red bean seeds, allows for the creation of new snacks with great consumer recognition. This suggests the possibility of their frequent consumption by potential consumers as a diet diversification or as an element enriching the diet [45].

To assess the biological role of the obtained plant snacks, an in vitro digestion process was carried out in a model of the digestive tract. The results obtained indicated that the digestion process affects the content of phenolic compounds and antioxidant activity at each stage of the digestion process. The recorded increase in the analyzed parameters in the final section of the digestive tract indicates the key role of intestinal microflora during this process. Plant-based snacks with added marjoram (RBM) are distinguished by the highest antioxidant activity and phenolic compound content at all stages of the digestion process, which may indicate its high health-promoting potential.

As shown by the research conducted by Wang et al. [46], fermentation of legumes can increase the release of polyphenols during the digestion process. They showed that fermentation significantly improved the availability of phenolic compounds in black bean tempeh, attributing this effect to the enzymatic activity of *Rhizopus oligosporus* during fermentation [46]. The enzymatic activity could have contributed to the breakdown of the cell walls of legumes, thus increasing the effect of releasing compounds during the digestion process [46].

The studies of Tarko et al. [47] also showed that the digestion of polyphenols begins in the stomach and duodenum, where they are decomposed and transformed. They noted that phenolic acids are particularly susceptible to degradation in these parts of the digestive tract, which leads to the formation of derivatives with high antioxidant potential. As a result of this process, polyphenols can change their chemical structure, which affects their biological activity. Moreover, the studies of Choi et al. [48] and Bezek and Maganja [49] indicate that certain intestinal bacteria, e.g., *Lactobacillus plantarum*, are very effective in converting polyphenols, thus increasing their bioactivity. In particular, quercetin and hesperidin were intensively metabolized by the intestinal microflora into more bioactive compounds [47,50,51]. This also suggests that the antioxidant activity of phenolic compounds may be increased by these processes and influence the composition of the gut microbiota [52,53].

The origin source of phenolic compounds also influences antioxidant activity and the ability of intestinal microflora to metabolize them [54,55,56,57]. Studies on various origin sources of phenolic compounds such as cocoa [54] and tea [55,56] have shown that polyphenols released from them can support the growth of *Bifidobacteria* and *Lactobacillus* bacteria. Moreover, the interaction between polyphenols and intestinal microbiota can lead to the production of secondary metabolites with increased antioxidant properties [58,59]. The diversity of intestinal microbiota is crucial for the effective metabolism of polyphenolic compounds, which may be of significant importance for human health, especially in the context of intestinal diseases, where polyphenols exhibit anti-inflammatory and antioxidant effects [60,61].

A similar relationship of increasing antioxidant activity was noted in the present study and depended on the type of digested snack variant. The greatest dynamics of changes in the digested content were noted in the case of the digestion of RBM and RBRB snacks. It should be noted, however, that various factors, such as temperature, time, and the type of processing, may also affect the content of polyphenols and their antioxidant activity [62], which is also indicated by the results obtained in the presented work. Similar conclusions can be drawn in the case of the determined contents of reducing substances and soluble proteins at individual stages of the digestion process, which indicates a close interdependence of the intestinal microbiome, its activity, and the plant matrix.

During the digestion process, the enzymatic activity of β-glucuronidase (EC 3.2.1.31) and β-glucosidase (EC 3.2.1.21) was also monitored. Both enzymes are produced by different strains of intestinal bacteria. Therefore, the state of the intestinal microbiota is crucial for the activity of this group of enzymes in the gastrointestinal tract [63]. Disturbance of the work of both enzymes, β-glucuronidase (hydrolysis of glucuronide bonds) and β-glucosidase (decomposition of complex carbohydrates), can lead to serious diseases of the body [59,64]. Some scientific reports suggest that a diet rich in biologically active compounds, especially phenolic compounds, may affect the intestinal microflora and, thus, enzymatic activity [65].

Similar conclusions can be drawn from the analysis of the results obtained in this study, where a decrease in enzymatic activity was noted, especially in the last section of the digestive tract, as well as the changes in the number of intestinal microflora. The obtained RBB, RBM, RBC, and RBRB snacks had a positive effect on the development of lactic acid bacteria, the best being the base snack (RBB), where the highest number of bacteria was noted, and on the other hand, the best potential antibacterial properties against pathogens such as *E. coli*—the snack with marjoram (RBM)—also had the highest content of phenolic compounds and activity after the digestion process.

Thus, the intestinal microflora is crucial for regulating the activity of these enzymes. β-glucosidase is a key enzyme for digesting plant fiber, produced by various types of bacteria, including *Lactobacillus* and *Bifidobacterium,* which are essential components of healthy intestinal microflora [66,67]. Moreover, Zhang et al. [66], in their studies, found that some *Lactobacillus* strains can inhibit the activity of alpha- and beta-glucosidases, which can lead to a decrease in blood glucose levels. In the case of β-glucuronidase (produced by *Enterococcus* spp., *E. coli*, *Clostridium* spp., or *Bacteroides* spp.), the dysfunction of intestinal microflora increases its activity, which can lead to an increase in the level of free estrogens and, consequently, to hormonal disorders and colon cancer [59,64].

## 5. Conclusions

Studies have shown that fermentation with lactic acid bacteria significantly improves the nutritional and functional properties of plant snacks based on fermented red bean seeds. Additives such as marjoram (RBM) had a significant effect on the content of phenolic compounds and their antioxidant activity and ultimately proved their functional and health-promoting properties. Snacks with the addition of beetroot (RBRB) and marjoram were rated the highest by consumers in terms of taste and general acceptance. The in vitro digestion processes of plant snacks indicated the significant role of intestinal microflora in the modeling of their biological activities. The highest content of phenolic compounds and their antioxidant activity were determined in the final stages of the digestion process. In addition, the RBM snack showed the best antibacterial properties, demonstrating an inhibitory effect on the growth of the *E. coli* pathogen. The conducted research confirmed that the biotechnological processing of red bean seeds with additives such as marjoram, beetroot, or carrot enables the creation of innovative products with high nutritional and health-promoting values that can be used in the daily diet. However, it needs to be clarified to what extent the compounds released in the digestive process will be bioavailable.

## Figures and Tables

**Table 1:** Chemical composition and bioactive components in the raw material during biotechnological treatment and in the products obtained from red bean seeds.

Products	Dry Matter (%)	Reducing Substance (mg·g^−1^ dm)	Soluble Protein (mg·g^−1^ dm)	Total Protein (mg·g^−1^ dm)	Protein Digestibility (%)	Total Polyphenols (mg GAE·g^−1^ dm)	Antioxidant Activity (mg TE·g^−1^ dm)
Red bean seeds during biotechnological processing: before and after the microbial fermentation process							
RB	88.24 ± 0.21 ^c^	7.08 ± 0.12 ^c^	82.09 ± 2.69 ^c^	225.70 ± 1.76 ^c^	29.04 ± 1.023 ^a^	4.53 ± 0.36 ^c^	10.24 ± 1.04 ^b^
BFRB	48.02 ± 0.40 ^a^	5.20 ± 0.29 ^a^	33.37 ± 2.69 ^b^	143.40 ± 0.14 ^a^	62.05± 1.018 ^b^	3.33 ± 0.02 ^a^	7.03 ± 0.68 ^a^
AFRB	50.27 ± 1.56 ^b^	5.60 ± 0.30 ^b^	15.76 ± 0.71 ^a^	146.40 ± 0.99 ^b^	80.97 ± 0.75 ^c^	3.76 ± 0.03 ^b^	12.11 ± 0.25 ^c^
Fermented red bean snacks with additives after the freeze-drying process							
RBB	90.42 ± 0.12 ^b^	5.10 ± 0.33 ^a^	17.16 ± 0.83 ^a^	242.31 ± 0.25 ^a^	80.37 ± 0.35 ^a^	2.92 ± 0.11 ^b^	7.31 ± 0.43 ^a^
RBM	91.01 ± 0.17 ^d^	9.39 ± 1.14 ^c^	21.03 ± 1.36 ^c^	258.20 ± 0.12 ^c^	82.62 ± 0.21 ^b^	3.66 ± 0.15 ^c^	10.63 ± 0.45 ^c^
RBC	89.54 ± 0.16 ^a^	23.91 ± 2.41 ^d^	18.68 ± 0.62 ^b^	255.01 ± 0.33 ^b^	81.15 ± 0.82 ^a^	2.10 ± 0.06 ^a^	8.36 ± 0.23 ^b^
RBRB	90.90 ± 0.14 ^c^	6.25 ± 0.19 ^b^	18.04 ± 0.50 ^a^	258.23 ± 0.89 ^c^	83.17 ± 0.65 ^b^	2.74 ± 0.09 ^b^	8.66 ± 0.75 ^b^

RB—raw red bean seeds; BFRB—red bean seeds after cooked before the fermentation process; AFRB—after the fermentation process; RBB—snacks with inulin 3%, salt 0.5% basic variant; RBM—snacks basic variant + marjoram (2%); RBC—snacks basic variant + carrot (30%); and RBRB—snacks basic variant + red beetroot (15%). Mean values with the same letters in the column (a–d) were not significantly different (α = 0.05).

**Table 2:** Chemical composition and bioactive components in the raw material.

Products	Dry Matter (%)	Reducing Substance (mg·g^−1^ dm)	Soluble Protein (mg·g^−1^ dm)	Total Protein (mg·g^−1^ dm)	Total Polyphenols (mg GAE g^−1^ dm)	Antioxidant Activity (mg TE g^−1^ dm)
Aquafaba	6.10 ± 0.05 ^a^	5.02 ± 0.15 ^a^	29.10 ± 0.33 ^b^	12.60 ± 0.12 ^a^	1.10 ± 0.05 ^a^	1.30 ± 0.06 ^a^
Marjoram (dried)	87.61 ± 0.37 ^d^	94.87 ± 3.35 ^c^	249.10 ± 5.38 ^d^	116.09 ± 0.58 ^d^	29.20 ± 068 ^b^	87.73 ± 6.37 ^d^
Carrot (raw)	13.71 ± 0.22 ^b^	170.22 ± 5.05 ^d^	82.69 ± 1.39 ^c^	14.85 ± 0.45 ^b^	1.37 ± 0.15 ^a^	5.32 ± 0.27 ^b^
Red Beetroot (cooked)	15.49 ± 0.44 ^c^	17.48 ± 0.34 ^b^	27.89 ± 0.67 ^a^	22.82 ± 0.05 ^c^	80.48 ± 0.70 ^c^	55.35 ± 4.32 ^c^

Mean values with the same letters in the column (a–d) were not significantly different (α = 0.05).

**Table 3:** The factor loadings for aroma and taste attributes of plant-based snacks (red bean wafers) with marjoram, carrot, or red beetroot and the control sample (without marjoram, carrot, or red beetroot).

Sensory Descriptors	Factor		
	F1	F2	F3
Aroma			
Essential oil	−0.991 *	−0.116	0.066
Herbal	−0.994 *	−0.102	−0.028
Starch	0.938 *	−0.088	0.335
Lemon	−0.172	−0.443	0.880 *
Bitter	0.088	0.993 *	0.072
Strange	0.376	−0.913 *	−0.160
Sour	−0.811 *	0.283	0.511
Fermentation	−0.849 *	−0.496	−0.183
Taste			
Essential oil	0.556	−0.793	0.250
Herbal	0.427	−0.864 *	0.265
Sour	0.421	0.906 *	−0.048
Salty	−0.295	−0.830 *	0.472
Sweet	−0.921 *	−0.355	0.161
Starch	−0.774	0.461	−0.434
Broth	−0.968 *	−0.237	0.079
Bitter	0.966 *	−0.241	−0.098
Strange	−0.006	−0.399	−0.917

Two factors (F1 and F2) were extracted by applying PCA to the mean values of descriptive sensory scores. Sensory attributes with numbers marked * are believed to be the most important.

**Table 4:** Correlation coefficients between the overall desirability and color, aroma, and taste desirability to plant-based snacks (red bean wafers) with marjoram, carrot, or beetroot and the control sample (without marjoram, carrot, or red beetroot).

Sensory Attributes	Consumer Desirability
Color desirability	0.720
Aroma desirability	0.611
Taste desirability	0.995
Texture desirability	0.532

**Table 5:** Changes in phenolic compounds and their activity during the in vitro digestion process.

The Stages of Digestion	Total Polyphenols (mg GAE g^−1^ d. c.)				Antioxidant Activity (mg TE g^−1^ d. c.)			
	RBB	RBM	RBC	RBRB	RBB	RBM	RBC	RBRB
before digestion	2.71 ± 0.12 ^dC^	2.85 ± 0.17 ^cD^	1.83 ± 0.05 ^bB^	1.29 ± 0.13 ^aA^	4.01 ± 0.13 ^bD^	5.92 ± 0.25 ^bC^	2.34 ± 0.15 ^aB^	1.99 ± 0.18 ^bA^
“stomach” after 2 h at pH 2.0	3.01 ± 0.29 ^eC^	3.27 ± 0.21 ^dC^	1.89 ± 0.12 ^bA^	2.33 ± 0.02 ^dB^	1.20 ± 0.11 ^aA^	5.17 ± 0.24 ^aD^	3.56 ± 0.24 ^bC^	1.54 ± 0.10 ^aB^
after “duodenum” pH 7.4	1.41 ± 0.12 ^aA^	1.82 ± 0.03 ^aB^	1.20 ± 0.16 ^aA^	2.13 ± 0.16 ^cB^	2.10 ± 0.09 ^cD^	5.88 ± 0.05 ^bC^	2.82 ± 0.05 ^aB^	1.94 ± 0.36 ^bA^
“small intestine” with fecal flora at pH 7.4	1.55 ± 0.09 ^aC^	1.91 ± 0.09 ^aC^	1.39 ± 0.04 ^aA^	1.51 ± 0.06 ^bB^	12.10 ± 0.01 ^dB^	14.09 ± 0.02 ^cC^	2.38 ± 0.42 ^aA^	2.10 ± 0.21 ^bA^
“small intestine” after 2 h at pH 7.4	1.90 ± 0.23 ^bC^	2.26 ± 0.04 ^bD^	1.32 ± 0.16 ^aA^	1.52 ± 0.05 ^bB^	15.20 ± 0.26 ^eC^	15.84 ± 0.24 ^dD^	4.38 ± 0.17 ^cB^	2.40 ± 0.03 ^cA^
“large intestine” at pH 8.0	2.23 ± 0.11 ^cB^	2.92 ± 0.32 ^cD^	1.91 ± 0.30 ^bA^	2.41 ± 0.09 ^eC^	19.10 ± 0.52 ^fC^	22.07 ± 0.45 ^eD^	5.62 ± 0.45 ^dB^	2.51 ± 0.17 ^cA^
“large intestine” after 18 h at pH 8.0	3.92 ± 0.33 ^fB^	5.61 ± 0.14 ^eD^	2.40 ± 0.01 ^cA^	5.02 ± 0.17 ^fC^	24.10 ± 0.65 ^gC^	28.82 ± 0.18 ^fD^	7.89 ± 0.44 ^eB^	5.35 ± 0.67 ^dA^

Mean values with the same letters in the column (a–g) were not significantly different (α = 0.05). Mean values with the same big letters in the row (A–D) were not significantly different (α = 0.05). RBB—snacks with inulin 3%, salt 0.5% basic variant; RBM—snacks basic variant + marjoram (2%); RBC—snacks basic variant + carrot (30%); RBRB—snacks basic variant + red beetroot (15%); d. c.—digestion content.

**Table 6:** Changes in reducing substance and soluble protein during the in vitro digestion process.

The Stages of Digestion	Reduce Substance (mg·g^−1^ d. c.)				Soluble Protein (mg·g^−1^ d. c.)			
	RBB	RBM	RBC	RBRB	RBB	RBM	RBC	RBRB
before digestion	6.70 ± 1.45 ^aA^	19.25 ± 0.95 ^aB^	31.15 ± 0.44 ^cC^	5.14 ± 0.23 ^aA^	17.14 ± 0.13 ^aD^	10.10 ± 1.06 ^aA^	16.71 ± 0.29 ^aC^	12.57 ± 0.53 ^aB^
“stomach” after 2 h at pH 2.0	11.39 ± 0.07 ^bA^	24.18 ± 0.07 ^bC^	46.72 ± 0.35 ^eD^	20.63 ± 0.89 ^cB^	53.30 ± 3.98 ^bC^	14.77 ± 0.22 ^bA^	64.42 ± 0.84 ^bD^	47.01 ± 0.70 ^bB^
after “duodenum” pH 7.4	55.39 ± 4.60 ^dB^	41.93 ± 4.31 ^cA^	43.50 ± 0.59 ^dA^	59.95 ± 0.52 ^eB^	89.20 ± 0.37 ^cD^	62.93 ± 1.05 ^cA^	85.82 ± 0.50 ^cC^	67.86 ± 1.36 ^cB^
“small intestine” with fecal flora at pH 7.4	59.57 ± 5.93 ^dC^	44.93 ± 5.09 ^cB^	46.21 ± 1.47 ^eB^	8.92 ± 0.66 ^bA^	96.29 ± 1.46 ^dC^	65.42 ± 0.88 ^dA^	95.38 ± 2.42 ^aC^	76.48 ± 1.21 ^dB^
“small intestine” after 2 h at pH 7.4	85.99 ± 9.57 ^eC^	80.62 ± 9.57 ^dC^	21.95 ± 0.78 ^aB^	9.55 ± 0.25 ^bA^	108.28 ± 1.34 ^eD^	71.49 ± 2.27 ^eA^	100.38 ± 3.17 ^dC^	79.80 ± 0.99 ^eB^
“ large intestine” at pH 8.0	97.16 ± 2.52 ^fC^	90.62 ± 2.32 ^dB^	25.10 ± 0.89 ^bA^	140.55 ± 5.09 ^fD^	329.98 ± 7.65 ^fD^	91.07 ± 1.45 ^fA^	123.62 ± 7.45 ^eC^	111.12 ± 3.17 ^fB^
“large intestine” after 18 h at pH 8.0	19.30 ± 1.40 ^cA^	49.31 ± 1.3 4 ^cC^	84.01 ± 1.11 ^dD^	23.30 ± 0.35 ^dB^	204.30 ± 6.75 ^gB^	162.78 ± 3.18 ^gA^	196.89 ± 4.84 ^fB^	166.35 ± 4.67 ^fA^

Mean values with the same letters in the column (a–g) were not significantly different (α = 0.05). Mean values with the same big letters in the row (A–D) were not significantly different (α = 0.05). RBB—snacks with inulin 3%, salt 0.5% basic variant; RBM—snacks basic variant + marjoram (2%); RBC—snacks basic variant + carrot (30%); RBRB—snacks basic variant + red beetroot (15%); d. c.—digestion content.

**Table 7:** The results of β-glucuronidase and β-glucosidase activity after snack digestion.

The Stages of Digestion	β-Glucuronidase Activity (U·g^−1^ d. c.)				β-Glucosidase Activity (U·g^−1^ d. c.)			
	RBB	RBM	RBC	RBRB	RBB	RBM	RBC	RBRB
“small intestine” after 2 h at pH 7.4	0.171 ± 0.012 ^cB^	0.188 ± 0.0014 ^cD^	0.146 ± 0.013 ^cA^	0.180 ± 0.013 ^cC^	0.132 ± 0.012 ^cA^	0.252 ± 0.025 ^cC^	0.180 ± 0.024 ^cB^	0.152 ± 0.010 ^cA^
“ large intestine” at pH 8.0	0.104 ± 0.008 ^bB^	0.165± 0.015 ^bD^	0.099 ± 0.002 ^bA^	0.147 ± 0.012 ^bC^	0.128 ± 0.013 ^bB^	0.223 ± 0.011 ^bC^	0.093 ± 0.012 ^bA^	0.125 ± 0.022 ^bB^
“large intestine” after 18 h at pH 8.0	0.067 ± 0.003 ^aB^	0.042 ± 0.001 ^aA^	0.060 ± 0.005 ^aB^	0.076 ± 0.08 ^aC^	0.087 ± 0.004 ^aA^	0.112 ± 0.008 ^aC^	0.089 ± 0.002 ^aA^	0.098 ± 0.004 ^aB^

Mean values with the same letters in the column (a–c) were not significantly different (α = 0.05). Mean values with the same big letters in the row (A–D) were not significantly different (α = 0.05). RBB—snacks with inulin 3%, salt 0.5% basic variant; RBM—snacks basic variant + marjoram (2%); RBC—snacks basic variant + carrot (30%); RBRB—snacks basic variant + red beetroot (15%); d. c.—digestion content.

**Table 8:** Changes in the intestinal microbiota during the digestion process of plant-based snacks.

The Stages of Digestion	RBB	RBM	RBC	RBRB
*Lactobacillus* (log10 cfu·mL−1)				
“small intestine” after 2 h at pH 7.4	7.24 ± 0.15 aA	7.48 ± 0.14 aA	7.44 ± 0.09 aA	7.34 ± 0.07 aA
“ large intestine” at pH 8.0	8.18 ± 0.04 bB	8.30 ± 0.02 aC	8.61 ± 0.07 bD	7.57 ± 0.05 bA
“large intestine” after 18 h at pH 8.0	11.70 ± 0.01 cC	10.16 ± 0.03 bA	10.26 ± 0.02 cB	10.27 ± 0.06 cB
*Bifidobacterium* (log10 cfu·mL−1)				
“small intestine” after 2 h at pH 7.4	7.22 ± 0.15 aA	7.30 ± 0.06 aA	7.54 ± 0.09 aB	7.33 ± 0.09 aA
“large intestine” at pH 8.0	8.31 ± 0.04 aB	8.36 ± 0.02 bB	8.64 ± 0.08 bC	8.11 ± 0.08 bA
“large intestine” after 18 h at pH 8.0	11.67 ± 0.06 bC	10.22 ± 0.06 cA	10.50 ± 0.07 cB	10.35 ± 0.11 cB
*Enterococcus* (log10 cfu·mL−1)				
“small intestine” after 2 h at pH 7.4	6.95 ± 0.15 aB	6.46 ± 0.11 aA	7.47 ± 0.05 aC	6.31 ± 0.08 aA
“ large intestine” at pH 8.0	8.24 ± 0.03 bB	7.18 ± 0.02 bA	8.61 ± 0.11 bC	7.05 ± 0.25 bA
“large intestine” after 18 h at pH 8.0	11.64 ± 0.12 cD	7.55 ± 0.01 cA	9.76 ± 0.15 cB	10.02 ± 0.07 cC
*Escherichia coli* (log10 cfu·mL−1)				
“small intestine” after 2 h at pH 7.4	6.17 ± 0.12 aA	6.68 ± 0.03 aB	7.07 ± 0.3 aC	6.36 ± 0.09 aA
“large intestine” at pH 8.0	6.35 ± 0.13 aA	6.60 ± 0.05 aB	7.47 ± 0.10 bC	7.63 ± 0.05 cD
“large intestine” after 18 h at pH 8.0	6.55 ± 0.14 aC	3.95 ± 0.07 bA	5.94 ± 0.09 cB	7.28 ± 0.07 bD

Mean values with the same letters in the column (a–c) were not significantly different (α = 0.05). Mean values with the same big letters in the row (A–D) were not significantly different (α = 0.05). RBB—snacks with inulin 3%, salt 0.5% basic variant; RBM—snacks basic variant + marjoram (2%); RBC—snacks basic variant + carrot (30%); RBRB—snacks basic variant + red beetroot (15%).

## Data Availability

The original contributions presented in this study are included in the article/Supplementary Material. Further inquiries can be directed to the corresponding author.

## Supplementary Materials

The following supporting information can be downloaded at: https://www.mdpi.com/article/10.3390/foods14010088/s1, Table S1: Mean scores of consumer desirability and profiling of plant-base snacks (red bean wafers) with marjoram, carrot, or red beetroot and the control sample (without marjoram, carrot, or red beetroot); Table S2: Correlations coefficients between the intensity of descriptors and consumer analysis parameters of plant-based snacks (red bean wafers) with marjoram, carrot, or red beetroot and the control sample (without marjoram, carrot or red beetroot); Table S3: Correlations coefficients between the intensity of descriptors (aroma and taste) and chemical parameters of plant-based snacks (red bean wafers) with marjoram, carrot, or red beetroot and the control sample (without marjoram, carrot, or red beetroot).
