# Supplementary material for: Innovative Application of Fermented Red Bean Seeds in Constructing Foods with Increased Biological Activity
Source: Foods. 2025 Jan 1;14(1):88. doi: 10.3390/foods14010088 (PMC11719576; doi:10.3390/foods14010088)
Supplement: Supplementary file 1 [file foods-14-00088-s001.zip › foods-3378059-supplementary.pdf]

Table S1. Mean scores of consumer desirability and profiling of plant-based snacks (red bean wafers) with marjoram, carrot or red beetroot and the control sample (without marjoram, carrot or red beetroot).

	Products			
	RBB	RBM	RBC	RBRB
**Consumer desirability**				
aroma	4.69±0.9[a]	4.42±1.2[a]	5.64±1.0[a]	6.14±1.2[a]
colour	6.29±0.7[a]	5.86±0.9[a]	5.86±0.8[a]	6.36±1.1[a]
taste	5.79±1.1[a]	5.80±0.8[a]	5.80±0.8[a]	7.07±0.7[a]
texture	5.06±0.6[a]	4.39±0.5[a]	5.79±1.0[a]	6.14±1.1[a]
overall	5.79±1.1[a]	5.80±0.9[a]	5.50±1.0[a]	7.00±0.8[a]
**Sensory profiling**				
Aroma				
essential oil aroma	1.75±0.2 [ba]	2.25±0.3 [a]	1.50±0.3 [b]	0.25±0.3 [c]
herbal aroma	1.50±0.2 [a]	1.75±0.2 [a]	1.25±0.2 [a]	0.20±0.1 [b]
starch aroma	1.00±0.2 [b]	1.25±0.3 [b]	1.20±0.3 [b]	2.50±0.3 [a]
lemon aroma	0.00±0.0 [a]	0.25±0.2 [a]	0.00±0.0 [a]	0.10±0.1 [a]
bitter aroma	0.50±0.4 [b]	0.60±0.3 [b]	1.25±0.2 [a]	0.75±0.2 [b]
strange aroma	0.90±0.3 [a]	0.80±0.4 [a]	0.60±0.3 [a]	0.90±0.3
sour aroma	1.00±0.5 [b]	1.75±0.2 [a]	1.50±0.3 [a]	0.75±0.3 [b]
fermentation aroma	2.00±0.3 [a]	1.90±0.3 [a]	1.00±0.2 [b]	0.50±0.2 [c]
Taste				
essential oil taste	0.20±0.1 [a]	2.50±0.2 [a]	0.70±0.3 [b]	0.50±0.2 [a]
herbal taste	0.50±0.2 [c]	2.75±0.2 [a]	1.00±0.2 [b]	1.10±0.2 [b]
sour taste	1.00±0.3 [a]	0.70±0.2 [a]	1.00±0.2 [a]	0.65±0.3 [a]
salty taste	0.80±0.2 [a]	1.00±0.3 [a]	0.90±0.2 [a]	1.00±0.2 [a]
sweet taste	0.00±0.0 [c]	0.25±0.3 [cb]	0.25±0.1 [b]	1.50±0.2 [a]
starch taste	1.20±0.3 [a]	0.80±0.2 [a]	1.00±0.2 [a]	1.25±0.2 [a]
broth taste	0.20±0.1 [a]	0.20±0.1 [a]	0.25±0.2 [a]	0.75±0.3 [a]
bitter taste	2.00±0.4 [ba]	2.75±0.4 [a]	1.75±0.2 [b]	0.75±0.2 [c]
strange taste	0.90±0.2 [a]	0.80±0.2 [ba]	0.40±0.2 [b]	0.75±0.2 [ba]

* Different letters within the same row represent significant differences (n=8) at a $\alpha \leq 0.05$

Table S2. Correlations coefficients between the intensity of descriptors and consumer analysis parameters of plant-based snacks (red bean wafers) with marjoram, carrot or red beetroot and the control sample (without marjoram, carrot or red beetroot).

Sensory descriptors	Consumer desirability	
	aroma	overall
Aroma		
essential oil	-0.916	-0.851
herbal aroma	-0.632	-0.878
starch aroma	0.978	0.957
lemon aroma	-0.576	0.180
bitter aroma	-0.253	-0.253
strange aroma	-0.277	0.631
sour aroma	0.764	-0.735
fermentation [illegible]	-0.814	-0.639
broth aroma	0.071	0.978
Taste		
essential oil	-0.264	-0.230
herbal	-0.121	-0.088
sour	-0.704	-0.731
salty	0.527	0.518
sweet	0.971	0.947
starch	0.597	0.588
broth	0.981	0.956
bitter	-0.812	-0.762
strange	0.216	0.313

Table S.3. Correlations coefficients between the intensity of descriptors (aroma and taste) and chemical parameters of plant-based snacks (red bean wafers) with marjoram, carrot or red beetroot and the control sample (without marjoram, carrot or red beetroot).

**Descriptors**	**Dray Matter (%)**	**Reducing Substance**	**Soluble**	**Protein**	**Total polyphenols**	**Antioxidant**
aroma						
essential oil	-0.09	0.15	0.52	-0.44	-0.27	0.33
herbal	-0.14	0.16	0.44	-0.52	-0.35	0.24
starch	0.44	-0.30	-0.14	0.78	0.56	0.10
lemon	0.75	-0.30	0.86*	0.73	0.62	0.95*
bitter	-0.80*	0.94*	0.01	-0.07	0.38	-0.08
strange	0.79	-0.99	-0.31	0.23	-0.30	-0.13
sour	-0.18	0.53	0.83*	-0.05	0.30	0.66
fermentation	0.15	-0.28	0.24	-0.50	-0.56	0.10
broth	0.43	-0.38	-0.28	0.69	0.42	-0.04
taste						
essential oil	0.44	0.03	0.98*	0.47	0.56	0.96*
herbal	0.52	-0.02	0.98*	0.59	0.65	0.99*
sour	-0.83*	0.46	-0.48	-0.97*	-0.72	-0.69
salty	0.53	-0.02	0.67	0.98*	0.95*	0.81*
sweet	0.41	-0.26	-0.14	0.78	0.57	0.09
starch	-0.02	-0.39	-0.93*	-0.14	-0.42	-0.80*
broth	0.36	-0.30	-0.29	0.68	0.45	-0.06
bitter	0.06	0.08	0.63	-0.29	-0.15	0.47
strange	0.79	-0.96*	-0.10	0.07	-0.40	0.01
